# Transcriptomic Analysis of Multipurpose Timber Yielding Tree *Neolamarckia cadamba* during Xylogenesis Using RNA-Seq

**DOI:** 10.1371/journal.pone.0159407

**Published:** 2016-07-20

**Authors:** Kunxi Ouyang, Juncheng Li, Xianhai Zhao, Qingmin Que, Pei Li, Hao Huang, Xiaomei Deng, Sunil Kumar Singh, Ai-Min Wu, Xiaoyang Chen

**Affiliations:** 1 State Key Laboratory for Conservation and Utilization of Subtropical Agro-bioresources (South China Agricultural University), Guangzhou, China; 2 Guangdong Key Laboratory for Innovative Development and Utilization of Forest Plant Germplasm, Guangzhou, China; 3 Guangdong Province Research Center of Woody Forage Engineering Technology, Guangzhou, China; 4 College of Forestry and Landscape Architecture, South China Agricultural University, Guangzhou, China; 5 Guangxi Botanical Garden of Medicinal Plants, Nanning, P.R. China; 6 Department of Botany, Faculty of Science, The MS University of Baroda, Vadodara, Gujarat, India; University of Western Sydney, AUSTRALIA

## Abstract

*Neolamarckia cadamba* is a fast-growing tropical hardwood tree that is used extensively for plywood and pulp production, light furniture fabrication, building materials, and as a raw material for the preparation of certain indigenous medicines. Lack of genomic resources hampers progress in the molecular breeding and genetic improvement of this multipurpose tree species. In this study, transcriptome profiling of differentiating stems was performed to understand *N*. *cadamba* xylogenesis. The *N*. *cadamba* transcriptome was sequenced using Illumina paired-end sequencing technology. This generated 42.49 G of raw data that was then *de novo* assembled into 55,432 UniGenes with a mean length of 803.2bp. Approximately 47.8% of the UniGenes (26,487) were annotated against publically available protein databases, among which 21,699 and 7,754 UniGenes were assigned to Gene Ontology categories (GO) and Clusters of Orthologous Groups (COG), respectively. 5,589 UniGenes could be mapped onto 116 pathways using the Kyoto Encyclopedia of Genes and Genomes (KEGG) pathway database. Among 6,202 UniGenes exhibiting differential expression during xylogenesis, 1,634 showed significantly higher levels of expression in the basal and middle stem segments compared to the apical stem segment. These genes included *NAC* and *MYB* transcription factors related to secondary cell wall biosynthesis, genes related to most metabolic steps of lignin biosynthesis, and *CesA* genes involved in cellulose biosynthesis. This study lays the foundation for further screening of key genes associated with xylogenesis in *N*. *cadamba* as well as enhancing our understanding of the mechanism of xylogenesis in fast-growing trees.

## Introduction

Wood is the most abundant biological resource on earth. It is an important raw material for lumber and paper manufacturing, and is increasingly being exploited as an environmentally cost-effective, renewable source for energy production [1]. Wood formation is an important plant developmental event resulting from the accumulation of secondary cell walls in the xylem. Wood formation or xylogenesis involves a sequence of developmental events at the cellular level including cell division, cell expansion, deposition of secondary cell walls, lignification and programmed cell death [2]. Secondary cell wall formation and lignification are critical steps in the maturation of xylem tracheary elements and fibre cells. The secondary cell wall is mainly composed of cellulose (40–50%), hemicellulose (~25%), and lignin (25–35%) as well as small amounts of pectin and protein [2].

Biofuel production has recently stimulated interest in understanding the biosynthesis of secondary cell walls, including its transcriptional regulation. For cellulose biosynthesis, both direct (sucrose synthase, SuSY) and indirect (invertase, INV) pathways produce UDP-glucose, which is the direct substrate for cellulose biosynthesis [3]. In *Arabidopsis thaliana*, cellulose synthases (CesA) form a complex in which AtCesA4, AtCesA7 and AtCesA8 are essential for secondary cell wall formation [4], whereas AtCesA1, AtCesA3 and AtCesA6 are involved in primary cell wall synthesis [5]. The biosynthesis of xylan, a major hemicellulose in the secondary cell wall, involves numerous glycosyltransferases (GTs). GT43 family members *IRX9*, *IRX9L*, *IRX14* and *IRX14L*, GT47 family members *IRX10*, *IRX10L*, *FRA8*/*IRX7* and *F8H*, and GT8 family members *IRX8*, *PARVUS*, *GUX1*, *GUX2* and *GUX4*, are all involved in xylan backbone elongation, synthesis of the reducing end tetrasaccharide sequence and addition of glucuronic acid or a 4-O-methylglucuronic acid branch to the xylan backbone [6–9]. Lignin is synthesized from the cinnamyl alcohol monomers p-coumaryl, coniferyl, and sinapyl, and these three monolignols are synthesized in the cytoplasm from phenylalanine using different biosynthetic enzymes [10, 11]. Additionally, caffeoyl shikimate esterase (CSE) hydrolyzes caffeoyl shikimate into caffeate and together with 4-coumarate: CoA ligase (4CL) in the lignin biosynthetic pathway, bypasses the second hydroxycinnamoyl-CoA: shikimate/quinate hydroxycinnamoyltransferase (HCT) reaction [12]. Also, there are several transcription factors that have been shown to regulate secondary cell wall biosynthesis in *A*. *thaliana* [13]. Some transcription factors, for example MYB103, are not only able to activate genes involved in cellulose biosynthesis, such as *CesA8*, but also lignin or xylan biosynthesis genes, such as *F5H*, especially in S lignin biosynthesis [14].

*Neolamarckia cadamba* (syn. *Anthocephalus chinensis*), a member of the Rubiaceae family, is widely distributed in South Asia and South China due to its high economic value [15]. To date, *N*. *cadamba* research has mainly focused on its medicinal value in the treatment of various ailments and extraction of bioactive compounds [16]. Among these, triterpenoid saponins are documented as important active components and their biosynthesis requires the mevalonate (MVA) pathway in the cytosol and the non-mevalonate or 2-C-methyl-D-erythritol4-phosphate/1-deoxy-D-xylulose 5-phosphate (MEP/DOXP) plastid pathway for backbone formation [17]. Subsequently, the backbone undergoes various oxidation, substitution, and glycosylation steps mediated by a series of enzymes including geranyl diphosphate synthase(GPPS), farnesyl diphosphate synthase (FPS), squalene synthase (SQS), squalene epoxidase (SQE), β-amyrin synthase (β-AS), cytochrome P450-dependent monooxygenases (CYP 450s) and glycosyltransferases (UGTs) [18]. However, there have been no studies to date that dissect the molecular mechanisms underlying the biosynthesis of these bioactive compounds in *N*. *cadamba*. Apart from medicinal utilization, *N*. *cadamba* wood is also a suitable alternative material for building, furniture, pulp production and biomass utilization in tropical and subtropical regions [19]. We recently studied *N*. *cadamba* as a model system to analyze heteroxylan formation at the biochemical and molecular level during wood formation. Microsomes isolated from the middle and basal stem region exhibited higher UDP-Xyl synthase and xylosyltransferase enzyme activity and higher gene expression related to heteroxylan biosynthesis compared to the apical part of the stem [20]. Despite this investigation, little is known about cell wall biosynthesis pathways and transcriptional regulatory networks involved in *N*. *cadamba* xylogenesis.

RNA sequencing (RNA-Seq) is a high throughput technology based on next-generation sequencing (NGS) that enables genetic studies of species without the requirement of corresponding sequenced reference genome information [21]. RNA-Seq has evolved in to a powerful method for transcriptome profiling due to its accuracy, high throughput nature and reproducibility [22]. Subsequently, it has dramatically improved the efficiency and cost effectiveness of gene discovery [23]. To better understand the molecular basis of wood formation in *N*. *cadamba*, we initiated a RNA-seq project (http://www.ncbi.nlm.nih.gov/bioproject/PRJNA232616) to analyze and characterize the transcriptomes of several classes of genes involved in wood formation. We hope this profiling study will help initiate a systematic breeding program geared towards genetically improving the quality and productivity of this multipurpose timber-yielding tree.

## Materials and Methods

### Plant materials

**Fig 1:**
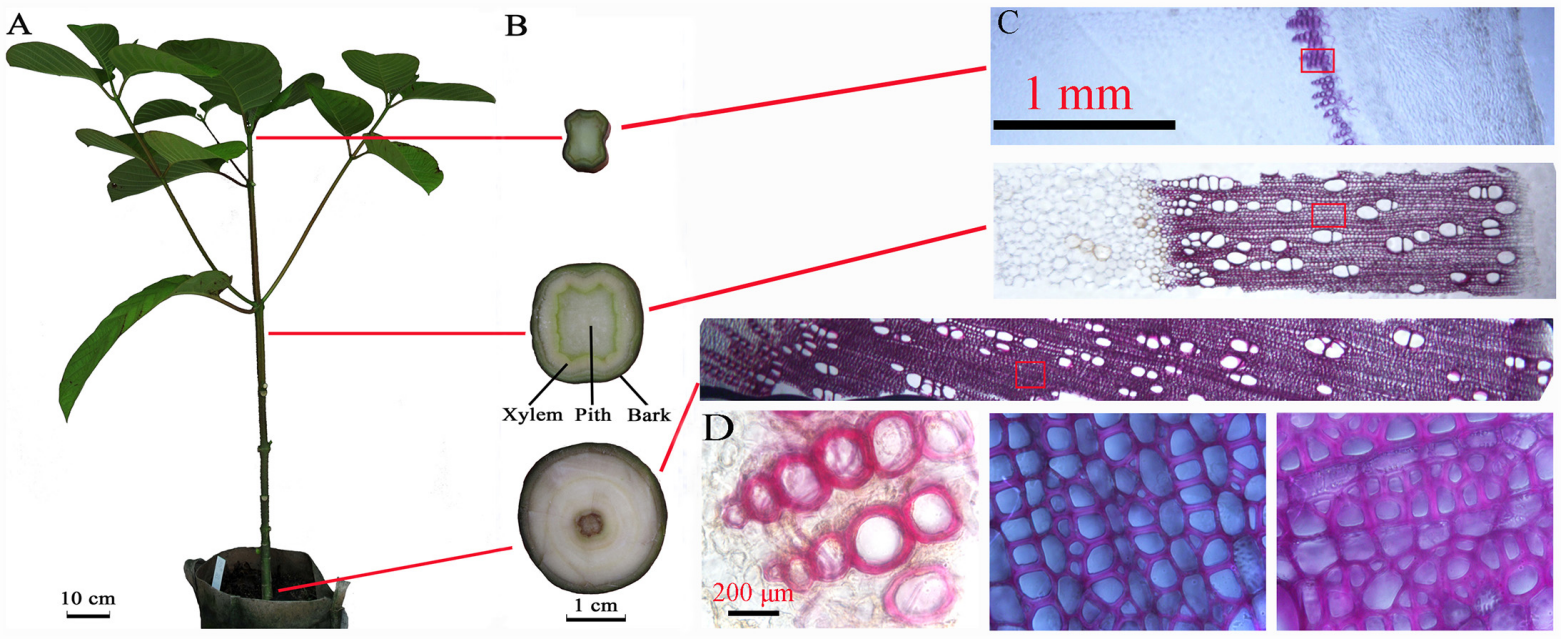
Tissues of *N*. *cadamba* used in deep sequencing. (A) The whole plant of greenhouse-grown *N*. *cadamba*. (B) The apical, middle and basal stem segments with bark from *N*. *cadamba*. (C) Phloroglucinol staining of transverse stem section. (D) Magnified view of red box region in C. Scale bar: A = 10 cm, B = 1 cm, C = 1 mm and D = 200 μm.

*N*.*cadamba* was grown as described previously [20]. Clones were proliferated and rooted as described previously [24] and grown in a greenhouse at 28/24°C, 14/10 h(day/night) and 330 μmol m^2^ s^1^ light. Peeled apical (just under apical bud, A), middle (M) and basal (just on soil surface, B) stem segments (1 cm in length) were sampled from a one-year-old plant, representing three different stages from primary to secondary growth, respectively (Fig 1). Each tissue was collected from two individual plants representing two biological replicates. All samples were quickly cut into pieces and immediately frozen in liquid nitrogen for RNA extraction.

### Sectioning of stems

Tissues from the three stem regions were fixed in FAA [5% (v/v) formalin, 5% (v/v) glacial acetic acid, 65% (v/v) ethylalcohol]. Next, tissues were embedded in 3% (w/v) agarose and sections of 40μm thickness cut using a Leica VT1000S vibratome fitted with a razor blade. Histochemical analysis using Wiesner and Maűle staining in Phloroglucinol-HCl was performed as previously described [25]. Stained stem sections were observed under an Olympus BX43F light microscope.

### RNA extraction, library construction and RNA-Seq

Total RNA from each sample was isolated using CTAB plus the OMEGA Plant RNA isolation kit as described previously [26]. Three libraries labeled A, M and B were constructed from RNA extracted from the apical, middle and basal stem segments, respectively. The RNA samples were checked for integrity on a 1.2% agarose gel and quantified using a Nanodrop 1000 spectrophotometer. RIN (RNA integrity number) values (> 8.0) of the samples were evaluated using an Agilent 2100 Bioanalyzer (Agilent Technologies, Santa Clara, CA, USA). Construction of the six libraries and RNA-Seq analysis were performed by Biomarker Biotechnology Corporation (Beijing, China). The mRNA enrichment and library construction were carried out according to protocol of NEB kit (E7490, E6110, E7500). Finally, the six libraries were sequenced using Illumina HiSeq^™^ 2000.

### *De novo* assembly

Clean reads were filtered from the raw reads by removing the adapter sequences and low quality sequences including reads with unknown nucleotides > 5% and more than 10% bases with a quality score (Q value) of < 20. The clean reads were then *de novo* assembled using the Trinity platform (http://trinityrnaseq.sourceforge.net/) with the parameters ‘K-mer = 25, group pairs distance = 300’ [21]. The reads obtained for the three stem samples and their two biological replicates were assembled together. Short reads were first assembled into longer contigs and then joined into transcripts based on the paired-end information and similarity between contigs. Finally, the longest transcript was taken as the sample UniGene. To facilitate access and utilization of the *N*. *cadamba* transcriptome sequencing data, all UniGene sequences have been uploaded to the Transcriptome Shotgun Assembly Sequence Database (TSA) at NCBI with accession numbers GASC01000001 to GASC01055370.

### Functional annotation

Putative functions of the UniGenes were annotated by carrying out a BLASTx analysis [27] with an E-value threshold of 10^−5^ against protein databases including the NCBI non-redundant (nr) database [27], the Swiss-Prot protein database [28], the Gene Ontology (GO) database [29], the Clusters of Orthologous Groups of Proteins (COG) database [30], and the Kyoto Encyclopedia of Genes and Genomes (KEGG) pathways database [31].

### Expression annotation

For each sample, the SOAPaligner (http://soap.genomics.org.cn/soapaligner.html) platform was used to map back reads to each UniGene. The number of mapped clean reads for each UniGene was counted and normalized into an RPKM value (reads per kb per million reads) [32]. The mean RPKM value for each UniGene from the biological replicates for each tissue was applied for downstream analysis. After that, differentially expressed genes (DEG) between samples were detected using DESeq software with a general chi-square test based on RPKM values. FDR (false discovery rate) was used to identify the P-value threshold in multiple tests in order to compute the significance of differences in each UniGene expression between two samples [33]. Genes were regarded as differentially expressed by the FDR < 0.01 and the absolute value of log_2_Ratio ≥ 1. In our study, DEGs between two samples were screened and used to compare GO classifications. Then, detailed expression profiles, I (up-regulation), II (irregularly regulated), III (irregularly regulated), IV (down-regulation) were distinguished for DEGs by using log_10_ RPKM_A_, log_10_ RPKM_M_ and log_10_ RPKM_B_ values, where I = (log_10_ RPKM_A_ ≤ log_10_ RPKM_M_ ≤ log_10_ RPKM_B_), II = (log_10_ RPKM_A_ ≤ log_10_ RPKM_M_ ≥ log_10_ RPKM_B_), III = (log_10_ RPKM_A_ ≥ log_10_ RPKM_M_ ≤ log_10_ RPKM_B_), and IV = (log_10_ RPKM_A_ ≥ log_10_ RPKM_M_ ≥ log_10_ RPKM_B_). UniGenes that were more abundantly expressed in libraries M and B compared to A were identified.

### Identification and regulation pathways analysis of cell wall-related transcription factors

A total of 82 transcription factors and transcriptional regulatory families of *A*. *thaliana* were downloaded from the PlnTFDB database [34]. UniGenes were searched against this database using the local NCBI-2.2.30+ BLASTx algorithm (E-value≤1E-10). Thetranscription factor KNAT7 was searched using local TBLASTN with the *A*. *thaliana* KNAT7 amino acid sequence (E-value≤1E-10) against the transcriptome library. UniGene sequences were double-checked by BLASTx searches against protein databases including the NCBI non-redundant (nr) database and the *A*. *thaliana* TAIR10 database.

According to the cell wall-related transcription factor regulatory network described by Schuetz et al [13] and Ohman et al [14], transcription factors involved in regulating lignin, cellulose and hemicellulose biosynthesis were identified in *N*. *cadamba* as described above. Relative (yellow—blue scale) and absolute (white—red scale) expression profiles of these genes were implicated in three different lignification processes represented by the A, M and B stem segments.

### Discovery of cellulose, mannan and monolignol biosyntheticgenes

Sequences of cellulose biosynthesis-related, mannan biosynthesis-related and monolignol biosynthesis-related proteins from *A*. *thaliana* were downloaded from the TAIR database (www.arabidopsis.org; shown in S1 File). The cellulose, mannan and monolignol biosynthesis-related structural genes were searched using TBLASTN with the amino acid sequences of the proteins (E-value ≤1E-10) against the transcriptomic library [27]. Then, these enzymes were identified in *N*. *cadamba* as previously described for KNAT7. According to the known cellulose [3], mannan [35, 36] and lignin [10–12] biosynthesis pathways, the UniGenes were ascribed to metabolic pathways.

### Discovery of cadambine, triterpenoidsaponin and phytosterol biosynthesis-related genes

Cadambine, triterpenoid saponin and phytosterol biosynthesis-related protein tryptophan synthase (TSA1-2: At3g54640, At4g02610; TSB1-2: At5g54810, At4g27070), tryptophan decarboxylase (TDC: X67662.1), geranyl diphosphate synthase (GPPS: AT2G34630), geraniol synthase (GES: JN882024.1), geraniol10-hydroxylase (G10H: KF561461.1), secologanin synthase (SLS: KF415117.1), strictosidine synthase (STR1-3: At1g74020, AT1G74020, At1g74000), farnesyl diphosphate synthase (FPS1-2: AT5G47770, AT4G17190), squalene synthase (SQS1-2:AT4G34640, AT4G34650), squalene epoxidase (SQE1-3: AT1G58440, AT2G22830, AT4G37760), β-amyrin synthase (β-AS: AT1G78950), and cycloartenolcyclase (CAS1: AT2G07050) from *A*. *thaliana* or *Catharanthus roseus* were downloaded from the TAIR database or Uniprot database [28]. The cadambine, triterpenoid saponin and phytosterol biosynthesis-related structural genes in *N*. *cadamba* were subsequently identified, allowing them to be assigned to metabolic pathways [18, 37].

### Real-time quantitative PCR analysis (RT-qPCR)

For RT-qPCR analysis, RNA samples were reverse transcribed into first-strand cDNA using PrimeScript^®^ RT Master Mix (Takara, China) according to the manufacturer’s protocol. The cDNA was diluted fifteen-fold and used as the template for RT-qPCR. Amplifications were carried out in triplicate in a total volume of 20μL containing 10μL of 2×SYBR^®^ Premix Ex Taq^™^ II (Takara, China), 2 μl of each primer (5 μM), 2 μl of cDNA, and 4 μl of ddH_2_O. Thermocycling conditions were as follows: an initial denaturation at 95°C for 30 s, followed by 40 cycles of 95°C for 5 s, 58°C annealing for 30 s and 72°C extension for 15 s, and an infinite hold at 10°C. The specificity of the PCR amplicon was checked using a heat dissociation protocol (from 65 to 95°C) after the final PCR cycle. The primers used in the RT-qPCR are shown in S2 File and the *cyclophilin* (JX902587) gene was used as the internal reference.

## Results

### Determination of cell wall components during xylogenesis

Previously, we analyzed *N*. *cadamba* cell wall composition during xylogenesis [20] and found that the levels of both lignin and non-cellulosic polysaccharides increased with stem maturity, exhibiting highest levels in the basal stem segment and lowest in the apical stem segment. The major non-cellulosic polysaccharide in the stem segments was heteroxylan with a substantially lower level of heteromannan. We also found that the proportion of cellulose decreased with stem maturity, with the highest level observed in the apical stem segment and the lowest in the middle segment, increasing in the basal stem segment. However, the proportion of lignin in both the middle and basal segments was significantly higher than in the apical stem segment [20]. As shown in histochemical analysis, lignin deposition was found only in vessel elements of the apical stem segments in contrast to the middle and basal stem segments that show lignin deposition in tracheary elements and fibers (Fig 1C and 1D).

### RNA-Seq and *de novo* assembly

To obtain a global overview of the *N*. *cadamba* transcriptome and gene expression during progression of xylogenesis in the stem, RNA was extracted from 1-year-old greenhouse grown plants initially raised from tissue culture. Three libraries (A, M, B) were constructed and RNA-Seq was performed using two biological replicates. RNA-Seq analysis generated 42.49G of raw data, and Q30 percentages (percentage of sequences with sequencing error rates < 0.1%) were found to be over 80% (Table 1). Based on the *de novo* assembly using Trinity [32], all clean short reads from the six libraries were assembled together into 5,870,723 contigs based on their overlap regions. The contigs were joined into 111,864 transcripts, and finally, a total of 55,432 UniGenes were identified with a mean length of 803.2bp and an N50 length of 1,501bp, among which 13,280 UniGenes (23.96%) were greater than 1 kb. The length distributions of contigs, transcripts and UniGenes are shown in Table 2. The data shows that the throughput and sequencing quality was high enough for further downstream analyses.

**Table 1:** RNA-Seq data summary for all samples.

Samples	BMK-ID	Total reads	Total bases (nt)	GC%	Q30%
Apical stem	A1^a^	25,085,358	5,066,725,234	46.03%	81.51%
segment	A2	32,126,330	6,489,002,687	44.08%	81.48%
Middle stem	M1	38,555,036	7,787,264,195	44.64%	82.13%
segment	M2	31,421,956	6,346,729,891	44.06%	81.26%
Basal stem	B1	47,999,394	9,695,200,117	44.29%	82.53%
segment	B2	35,170,009	7,103,808,681	43.95%	82.14%
Total		210.39M	42.49G		

^a^ biological replicates

**Table 2:** Length distribution of assembled contigs, transcripts, UniGenes and predicted ORFs of UniGenes.

Length range	Contigs	Transcripts	UniGenes	ORF
200–300	5,820,454(99.14%)	21,414(19.14%)	17,894(32.28%)	38,179(69.20%)^a^
300–500	21,669(0.37%)	19,663(17.58%)	14,433(26.04%)	4,556(8.26%)
500–1000	14,456(0.25%)	21,046(18.81%)	9,825(17.72%)	5,443(9.87%)
1000–2000	9,122(0.16%)	27,098(24.22%)	7,816(14.10%)	5,177(9.38%)
2000+	5,022(0.09%)	22,643(20.24%)	5,464(9.86%)	1,816(3.29%)
Total number	5,870,723	111,864	55,432	55,171
Total length	269,782,291	137,157,312	44,522,744	23,683,593
N50 length	44	2,023	1,501	1,086
Mean length	45.95	1226.11	803.20	429.28

^a^ The length range is <300 bp

### Functional annotation

Approximately 47.8% of the UniGenes (26,487) were annotated by BLASTx, with a threshold of 10^−5^, using five public databases (NCBI non-redundant (nr) database, Swiss-Prot protein database, Gene Ontology (GO) database, Clusters of Orthologous Groups of Proteins (COG) database and the Kyoto Encyclopedia of Genes and Genomes (KEGG) database). One half (12,293) of the UniGenes had a length of > 1000 bp. The functional annotation distributions of UniGenes are shown in Table 3.

**Table 3:** Summary for UniGene functional annotation.

Annotated databases	All sequence	> = 300 bp	> = 1000 bp
COG	7,754	7,093	5,255
GO	21,699	18,499	11,022
KEGG	5,589	4,780	3,042
Swissprot	19,590	16,967	10,559
nr	26,404	22,213	12,290
All	26,487	22,267	12,293

**Fig 2:**
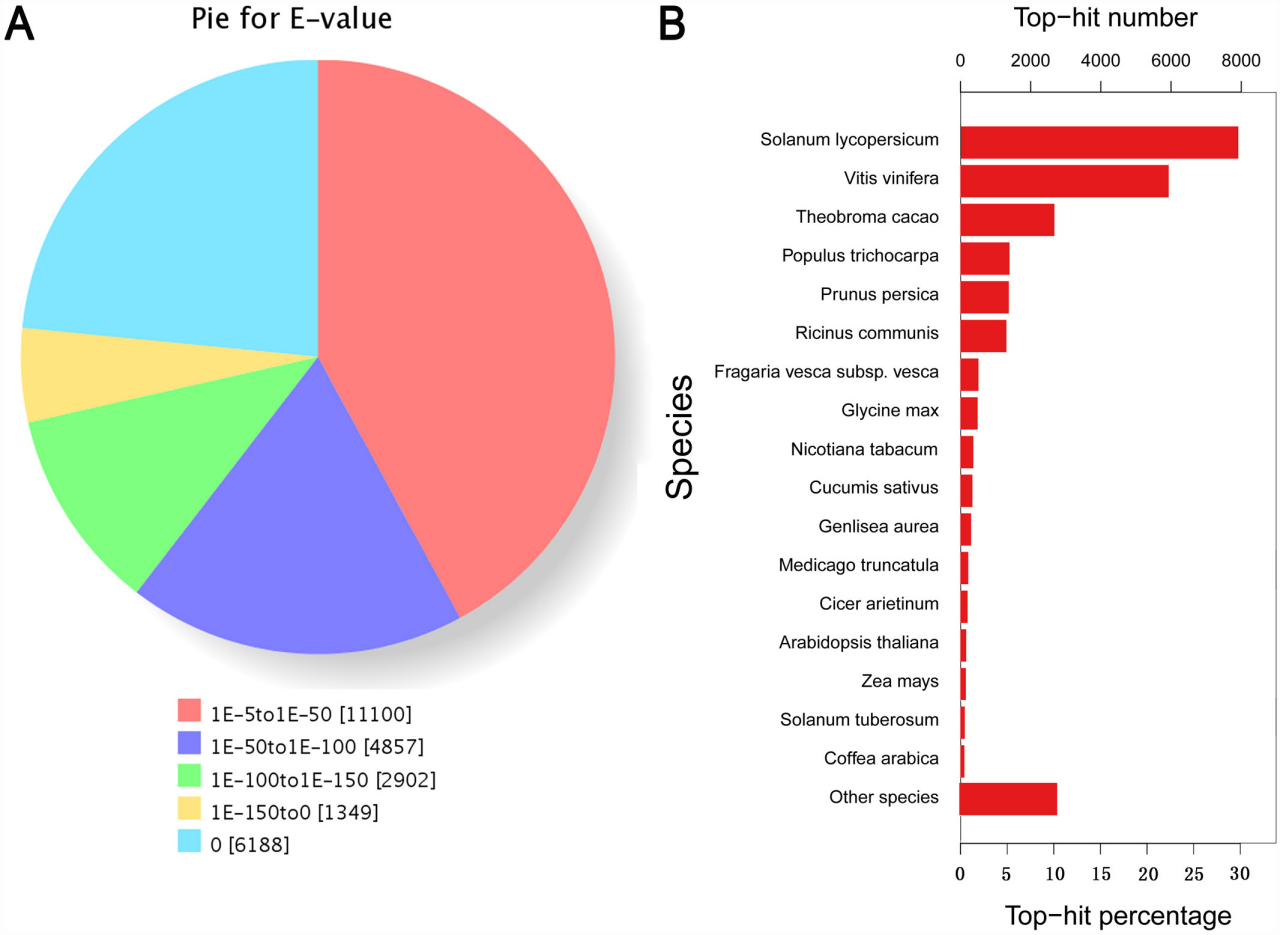
Characteristics of the homology search of *N*. *cadamba* UniGenes in the nr database. (A) E-value distribution of the top BLASTx hits against the nr database for each UniGene. (B) Number and percentage of UniGenes matching the 17 top species using BLASTx in the nr database.

Based on nr annotation and the E-value distribution, 57.9% of UniGenes showed a very strong homology (E-value < 10^−50^) to available plant sequences (Fig 2A). The 17 top-hit species based on nr annotation are shown in Fig 2B. Nearly 73% of UniGenes could be annotated with sequences from the 5 top-hit species (Fig 2B, S3 File), which were *Solanum lycopersicum*, *Vitis vinifera*, *Theobroma cacao*, *Populus trichocarpa* and *Prunus persica*.

Generally, Gene Ontology (GO) analysis was carried out to classify the functions of the assembled UniGenes in terms of their associated biological processes, cellular components, and molecular functions [29]. To better understand functional categories, GO analysis was employed to annotate UniGenes by known proteins using the Blast2GO program [38], after which the GO functional classifications of these UniGenes were performed using WEGO software [39]. A total of 21,699 UniGenes were classified according to the three GO categories. Under the biological process category, large numbers of UniGenes were categorized as cellular process (17,099, 78.8%) and metabolic process (16,414, 75.6%). Within the cellular component category, large numbers of UniGenes were categorized as cell part, cell and organelle component categories. As for the molecular function category, binding (13,353, 61.5%) and catalytic activity (11,293, 52.0%) were the most highly represented categories (S1 Fig, S4 File). Furthermore, a total of 205, 165, 337, 61and 19 UniGenes were annotated within the cellulose biosynthetic process (GO:0030244), lignin biosynthetic process (GO:0009809), xylan biosynthetic process (GO:0045492), glucuronoxylan biosynthetic process (GO:0010417) and mannan synthase activity (GO:0051753), respectively (S5 File).

The COG protein database is an attempt on phylogenetic classification of the proteins encoded in complete genomes of species [40]. All UniGenes were subjected to a search against the COG database for functional prediction and classification resulting in the assignment of 7,754 UniGenes. The COG database represented major phylogenetic lineages of *N*. *cadamba*, as shown in S2 Fig and S6 File. According to the COG annotation, these UniGenes were classified into 25 different functional classes, with the largest cluster being general function prediction only (group R, 2,177, 28.1%) followed by replication, recombination and repair (group L, 1,154, 14.9%). However, no UniGene was assigned to extracellular structures (group W). It was noteworthy that there were 618, 375 and 263 UniGenes sharing homology with carbohydrate transport and metabolism (group G), secondary metabolites biosynthesis, transport and catabolism (group Q) and cell wall/membrane/envelope biogenesis (group M), respectively, including UniGenes related to cell wall biosynthesis. This data will be useful in exploring protein classification and evolution rates [41].

By mapping to the KEGG reference pathways, 5,589 UniGenes were assigned to116 pathways (S7 File) in the KEGG database [31]. Ribosome pathways (Ko03010) were the most enriched (201), followed by plant hormone signal transduction (Ko04075, 190). Moreover, 79 UniGenes were mapped to phenylpropanoid biosynthesis pathways (Ko00940), with a majority of genes participating in lignin biosynthesis, and 146 UniGenes were mapped to starch and sucrose metabolism (ko00500), with a majority of genes participating in cellulose, mannan and heteroxylan biosynthesis (S8 File).

### Differentially expressed genes in different stem segments

**Fig 3:**
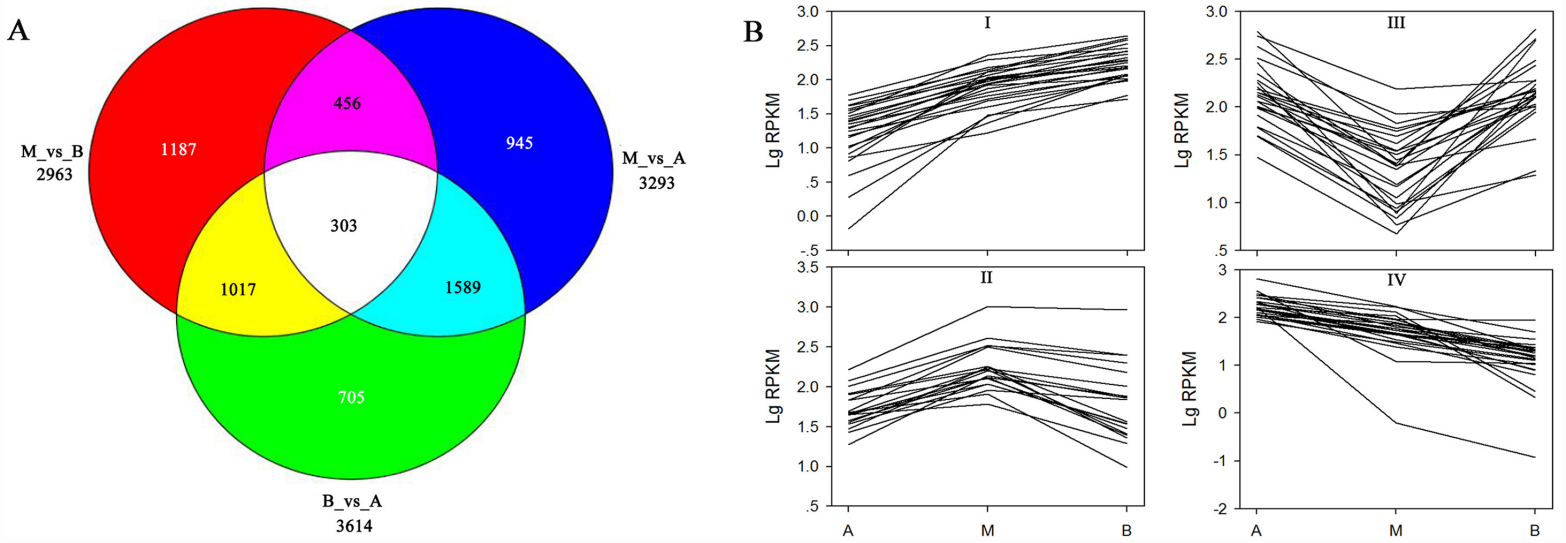
DEGs Venn diagram and expression profile. (A) Venn diagram of DEGs. A, apical stem segment; M, middle stem segment; B, basal stem segment. (B) Four expression profiles of DEGs. I and IV indicate UniGenes which are up-regulated and down-regulated, respectively, and II and III indicate those with irregular expression. Twenty-five UniGenes were selected for the diagram for each expression profile.

Differentially expressed genes (DEGs) among the three different stem segments were identified by DESeq software [33]. Based on this analysis, a total of 6,202 UniGenes were identified as DEGs in at least two libraries (Fig 3A, S9 File). Among these UniGenes, 3,293, 3,614 and 2,963 showed differential expression between A and M, A and B, M and B, respectively (Fig 3A, S1 Table). GO and COG classification is shown in S1 and S3 Figs respectively. Amongst the DEGs, 4 groups were defined according to their variations in expression profiles, containing 1,119, 1,436, 1,588 and 2,059 UniGenes, respectively. Group I was defined as being up-regulated, group IV as down-regulated, and groups II and III had irregular expression patterns (Fig 3B). Each of these four groups is shown in S10 File. Moreover, there were 1,634 UniGenes with a higher expression profile in the M and B libraries compared to the A library, and these UniGenes are shown in S9 File.

Among the DEGs and 1,634 UniGenes, there were 25 and 10 UniGenes annotated in the cellulose biosynthetic process (GO:0030244) category, 42 and 23 in the lignin biosynthetic process (GO:0009809), 74 and 31 in the xylan biosynthetic process (GO:0045492), 11 and 5 in the glucuronoxylan biosynthetic process (GO:0010417), and 10 and 0 in the mannan synthase activity (GO:0051753) category, respectively (S11 File).

### Transcription factors related to cell wall component biosynthesis

A total of 1,782 *N*. *cadamba* UniGenes with high sequence identity (E-value ≤ 1E-10) corresponding to 80 out of 82 *A*. *thaliana* transcription factor families downloaded from PlnTFDB were found in the stem libraries. The five most abundant transcription factor gene families were bHLH, C3H, C2H2, MYB and the HB group (S12 File). These are mainly associated with plant growth, development, stress responses, cell differentiation, morphogenesis, RNA metabolism, secondary cell wall formation and secondary metabolism [42–46].

Secondary cell wall formation is a critical step in the maturation of tracheary elements and fibre cells in the xylem [6, 47] and regulated by MYB [14, 48–54] and NAC [55–64] transcription factor families. In this study, 77 and 45 UniGenes were found with high sequence identity (E-value ≤ 1E-10) corresponding to the *A*.*thaliana* MYB and NAC transcription factor families, respectively (S12 File). Of 77 MYB transcription factor UniGenes, 28 UniGenes belonged to DEGs among the A, M and B libraries, including 12 UniGenes with an expression profile where the abundance in the M and B libraries was higher than in the A library. Of the 45 NAC transcription factor UniGenes, 19 UniGenes, including 12 UniGenes with the same expression profile as above, were identified as DEGs.

**Fig 4:**
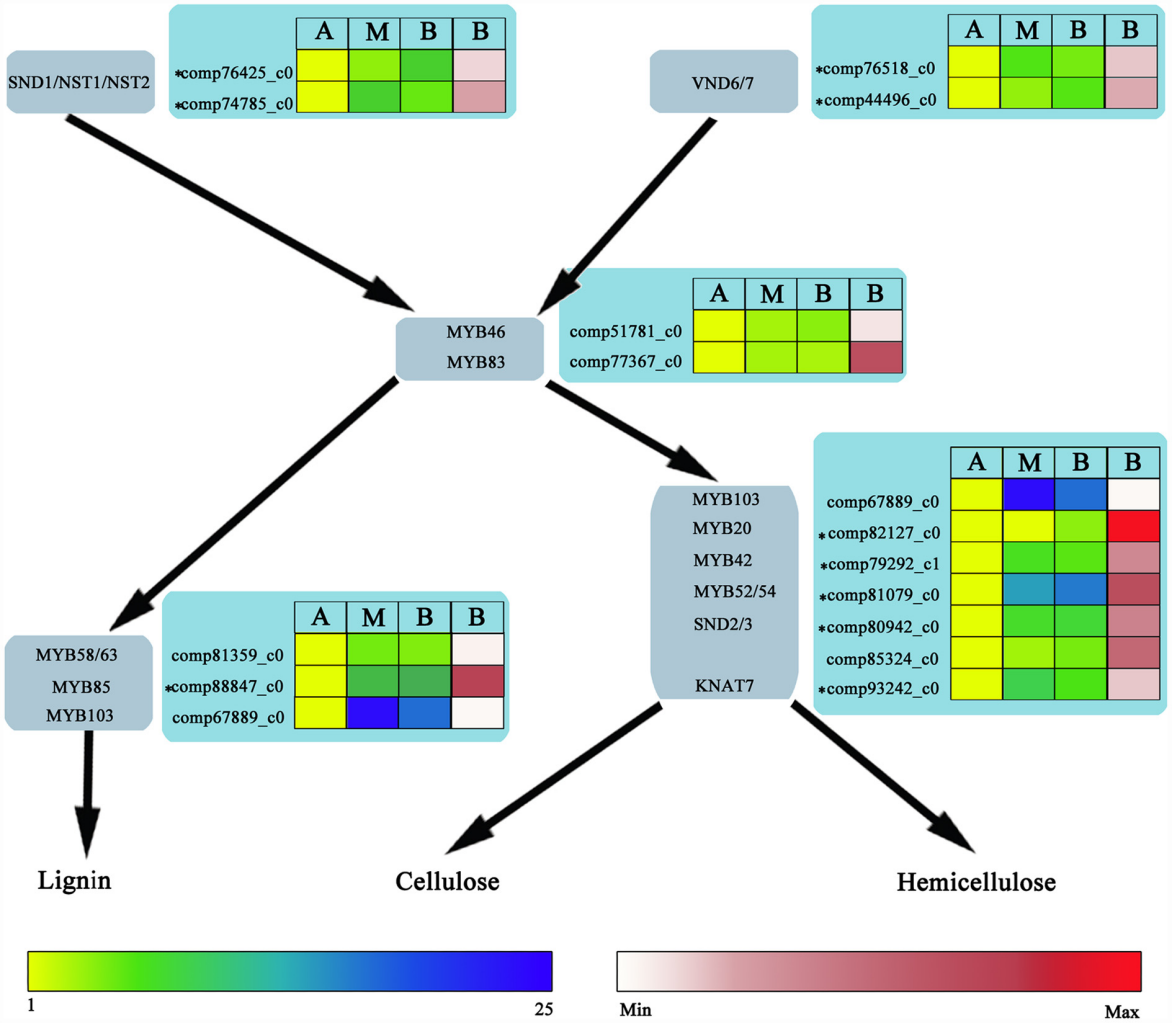
*N*. *cadamba* UniGenes in the transcriptional network regulating secondary cell wall (SCW) biosynthesis according to *A*. *thaliana*. Light grey boxes indicate major transcription factors of the Arabidopsis transcriptional network leading to biosynthesis of the three major SCW constituents. Light blue boxes indicate *N*. *cadamba* putative UniGenes encoding orthologues. * indicates the DEGs. Yellow-blue scale and white-red scale indicate relative and absolute expression profiles, respectively. A, apical stem segment; M, middle stem segment; B, basal stem segment. Absolute expression level (RPKM) is only shown for the basal stem segment.

A network of transcription factors involved in the biosynthesis of the secondary cell wall has been identified in *A*. *thaliana* [13, 14, 47, 52, 57, 58]. Based on this network, UniGene transcription factor sequences identified in this study were double-checked by BLAST searches against protein databases including the NCBI nr database and the *A*. *thaliana* TAIR10 database. Candidate genes corresponding to most of the known transcription factors involved in regulatory networks in *A*. *thaliana* have been found in *N*. *cadamba* (Fig 4). Moreover, most of them were identified as DEGs and had an expression profile with higher expression abundance in the M and B libraries than in the A library (Fig 4, S12 File), including UniGenes comp51781_c0 (MYB46), comp77367_c0 (MYB83), comp67889_c0 (MYB103), comp81359_c0 (MYB56/63) and comp85324_c0 (SND2/3), despite no significant differences observed.

### Genes involved in the cellulose and mannan biosynthetic pathways

**Fig 5:**
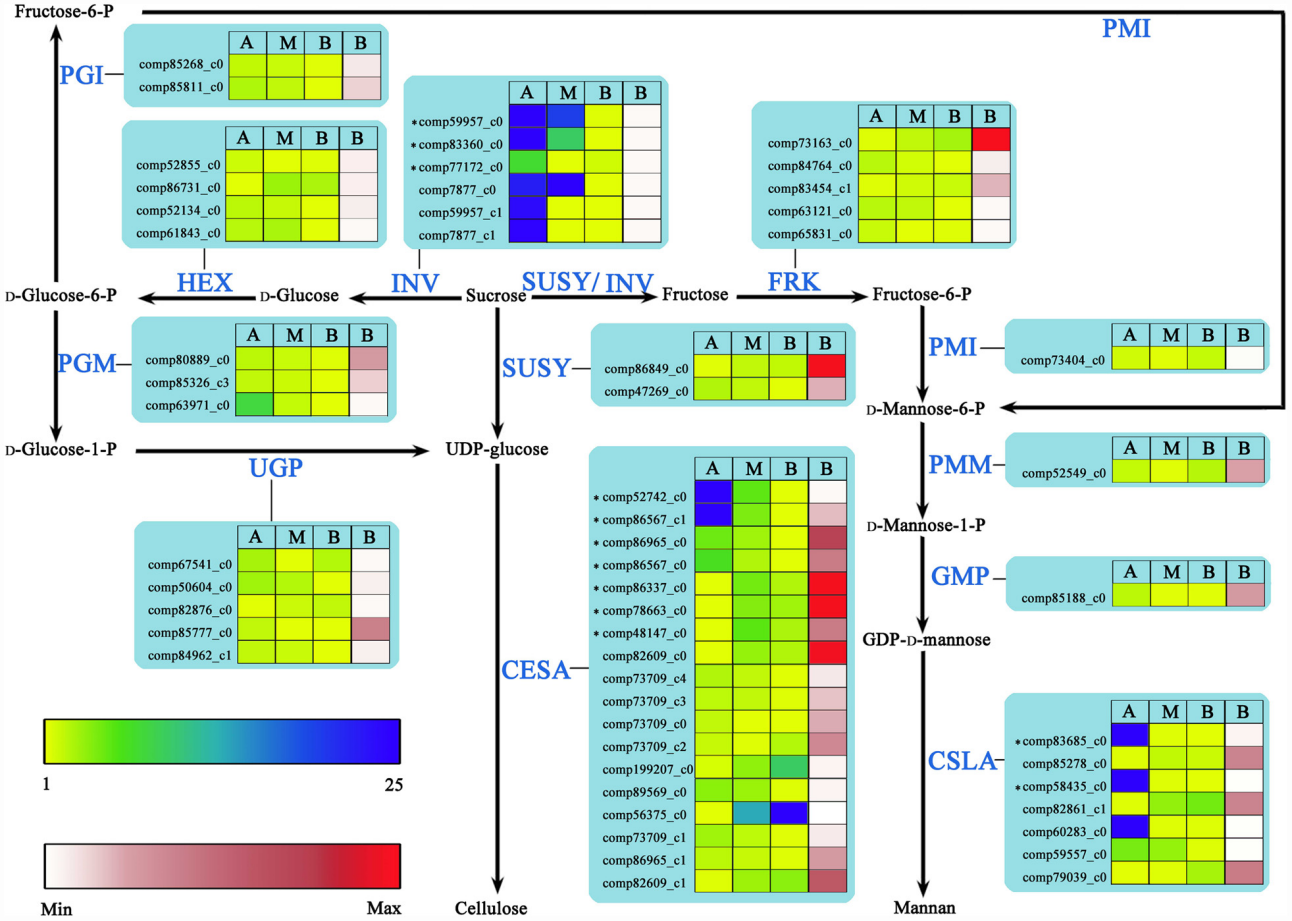
UniGenes involved in cellulose and mannan biosynthesis in wood-forming tissues of *N*. *cadamba*. Sugar and polymer intermediates are shown in black, whereas the proteins (enzymes) involved in each step are shown in blue. Detailed protein names, annotation and RNA-Seq expression data are provided in S12 File. * indicates the DEGs. Yellow-blue scale and white-red scale indicate relative and absolute expression profiles, respectively. A, apical stem segment; M, middle stem segment; B, basal stem segment. Absolute expression level (RPKM) is only shown for basal stem segment. CESA, cellulose synthase; CSLA, cellulose synthase like A; FRK, fructokinase; GMP, GDP-D-mannose pyrophosphorylase; HEX, hexokinase; INV, invertase; PGI, phosphoglucose isomerase; PGM, phosphoglucomutase; PMI, phosphomannose isomerase; PMM, phosphomannomutase; SUSY, sucrose synthase; UGP, UDP-glucosepyrophosphorylase.

Sucrose catabolism produces both direct and indirect substrates for cellulose biosynthesis, and indirect substrates for mannan biosynthesis *in planta*, which means that cellulose and mannan biosynthetic pathways converge on sucrose [3, 36]. Most of the UniGenes encoding enzymes participating in cellulose or mannan biosynthetic pathways were not identified as DEGs. However, the majority of these UniGenes had a higher expression abundance in apical stem segments compared to the middle and basal stem segments, including UniGenes comp86965_c0 showing 82% identity with AtCesA1, and comp52742_c0, comp86567_c0 and comp86567_c1 showing high identity with AtCesA2/6/9 by BLASTx. In contrast, the UniGenes comp86337_c0 (82% identity with AtCesA7 by BLASTx) and comp78663_c0 (73% identity with AtCesA4 by BLASTx) exhibited opposite expression pattern (Fig 5, S13 File).

### Genes involved in the monolignol biosynthetic pathway

**Fig 6:**
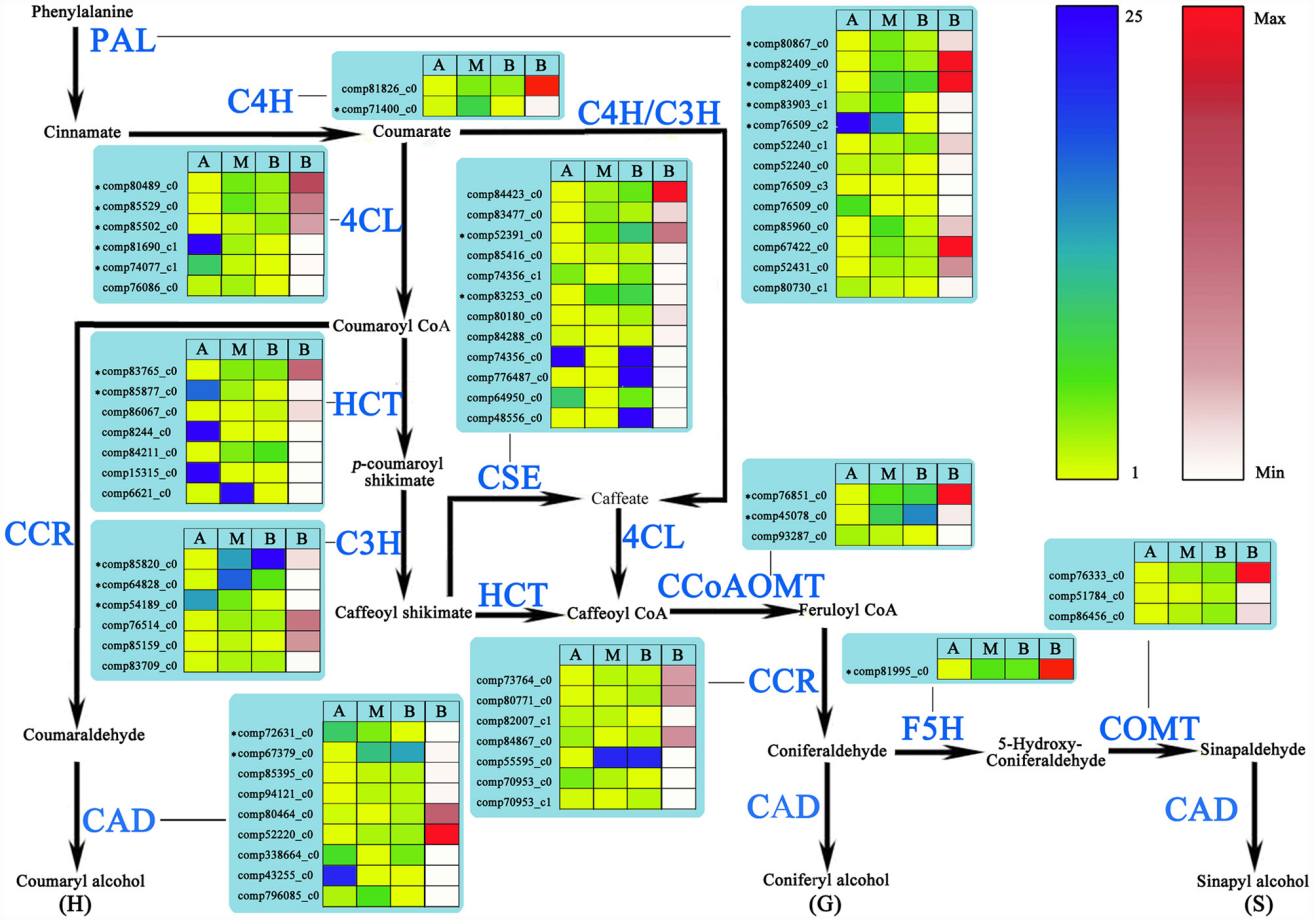
UniGenes involved in lignin biosynthesis in wood forming tissues of *N*. *cadamba*. Phenylalanine, intermediates and monolignols are shown in black, while the proteins (enzymes) involved in each step are shown in blue. Detailed protein names, annotation and RNA-Seq expression data are provided in S13 File. * indicates the DEGs. Yellow-blue scale and white-red scale indicate relative and absolute expression profiles, respectively. A, apical stem segment; M, middle stem segment; B, basal stem segment. Absolute expression level (RPKM) is only shown for basal stem segment. 4CL, 4-coumarate: CoA ligase; C3H, p-coumarate 3-hydroxylase; C4H, cinnamate 4-hydroxylase; CAD, cinnamyl alcohol dehydrogenase; CCoAOMT, caffeoyl-CoA O-methyltransferase; CCR, cinnamoyl-CoA reductase; COMT, caffeic acid O-methyltransferase; CSE, Caffeoylshikimate esterase; F5H, ferulate 5-hydroxylase; HCT, p-hydroxycinnamoyl-CoA: quinate shikimate p-hydroxycinnamoyltransferase; PAL, phenylalanine ammonia-lyase.

The main monolignols of lignin are coumaryl, coniferyl and sinapyl alcohols, which are finally incorporated into the lignin polymer as p-hydroxyphenyl (H), guaiacyl (G) andsyringyl (S) units, respectively [11]. In this study, a total of 69 UniGenes were found in *N*. *cadamba* transcriptomes with an E-value ≤ 1E-10 corresponding to all eleven monolignol biosynthesis enzymes in *A*. *thaliana* (Fig 6). The expression of 1–3 members of each gene family (PAL, 4CL, HCT, C3H, CSE, CCoAOMT, F5H and CAD) increased in both M and B libraries as compared to the A library (Fig 6, S14 File). Additionally, compared with the number of genes that encode each one of the eleven key enzymes involved in the lignin biosynthesis pathway in the *A*. *thaliana* TAIR database, there are more UniGenes in *N*. *cadamba* and genes in the *Populus trichocarpa* [65] and *Eucalyptus grandis* [3] genomes (Table 4).

**Table 4:** Comparison in number of genes that encode eleven key enzymes in the monolignol biosynthesis pathway among *A*. *thaliana*, *N*. *cadamba*, *P*. *trichocarpa* and *E*. *grandis*.

Enzymes	*A*. *thaliana*	*N*. *cadamba*	*P*. *trichocarpa*	*E*. *grandis*
PAL	4	13	5	9
C4H	1	2	3	2
4CL	4	6	17	13
CCR	2	7	9	9
CAD	9	9	16	46
HCT	1	7	7	5
C3H	1	6	4	4
CSE	1	12	2	4
CCoAOMT	1	3	6	17
COMT	1	3	25	67
F5H	1	1	3	2

### Genes involved in the cadambine, triterpenoid saponin and phytosterol biosynthetic pathways

**Fig 7:**
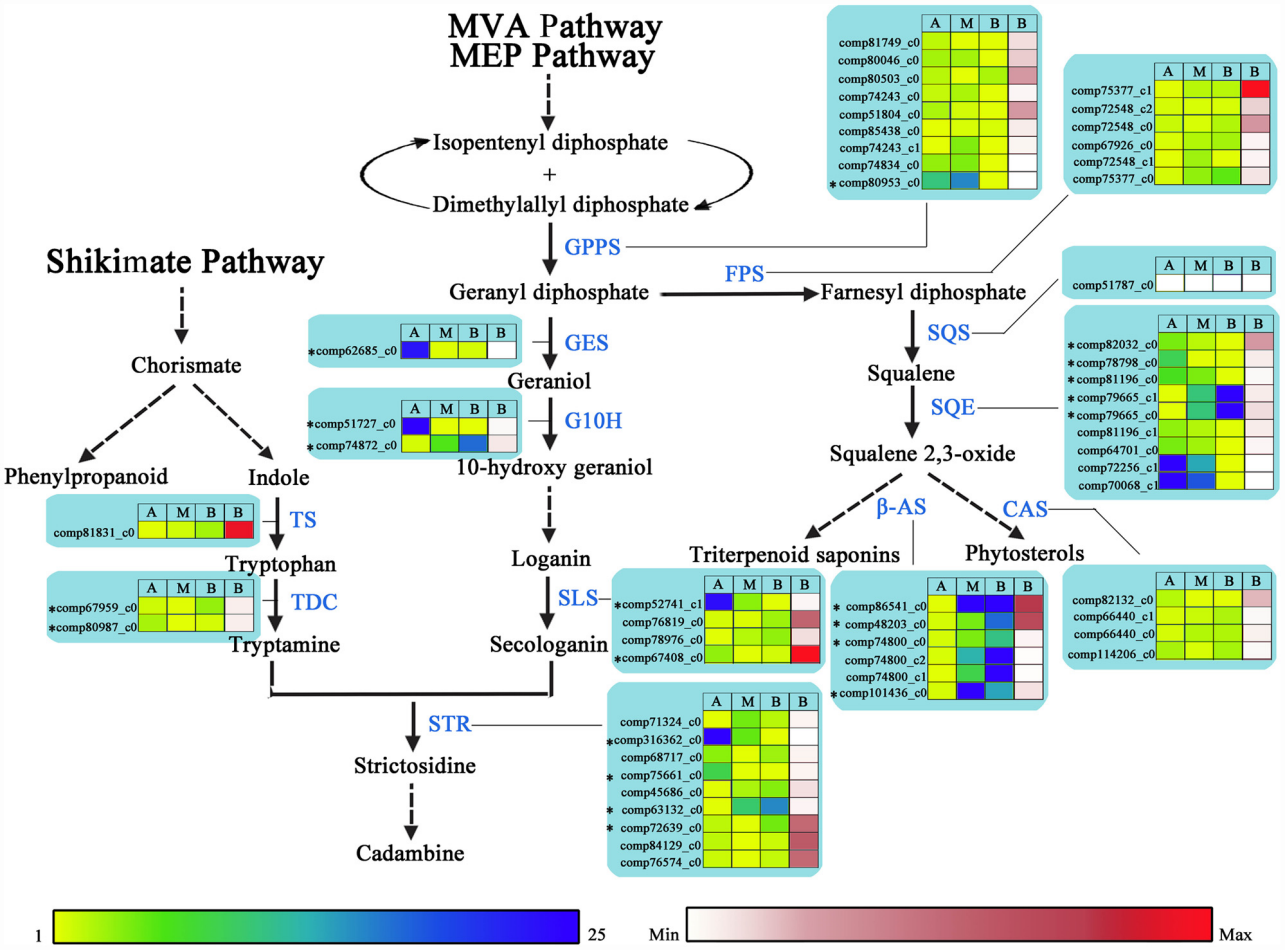
UniGenes involved incadambine, triterpenoid saponin and phytosterol biosynthesis. Intermediates, cadambine, triterpenoid saponins and phytosterols are shown in black, whereas the proteins (enzymes) involved in each step are shown in blue. Detailed protein names, annotation and RNA-Seq expression data are provided in S15 File. * indicates the DEGs. Yellow-blue scale and white-red scale indicate relative and absolute expression profiles, respectively. A, apical stem segment; M, middle stem segment; B, basal stem segment. Absolute expression level (RPKM) is only shown for basal stem segment. CAS, Cycloartenol cyclase; FPS, farnesyl diphosphate synthase; G10H, geraniol 10-hydroxylase; GES, geraniol synthase; GPPS, geranyl diphosphate synthase; SLS, secologanin synthase; SQE, squalene epoxidase; SQS, Squalene synthase; STR, strictosidinesynthase; TDC, tryptophan decarboxylase; TS, tryptophan synthase; β-AS, β-amyrin synthase.

Medicinal ingredients isolated from *N*. *cadamba* plants have traditionally been used in the treatment of various human ailments, such as diabetes mellitus, wounds and fever, as well as for their antimicrobial activity and antitumor properties [16]. Medicinal properties of *N*. *cadamaba* might be due to the presence of bioactive compounds such as alkaloids, triterpenoids and iridoids in plant tissues [66–68]. In the present study, a total of 54 UniGenes were found in the *N*. *cadamba* transcriptomes with E-values≤ 1E-10 corresponding to all eleven enzymes in *A*. *thaliana* or *C*. *roseus* (Materials and methods), except for the UniGene comp51787_c0, which was found in the transcriptomes from the other tissues sample (Fig 7, S15 File).

### Expression analysis by RT-qPCR

**Fig 8:**
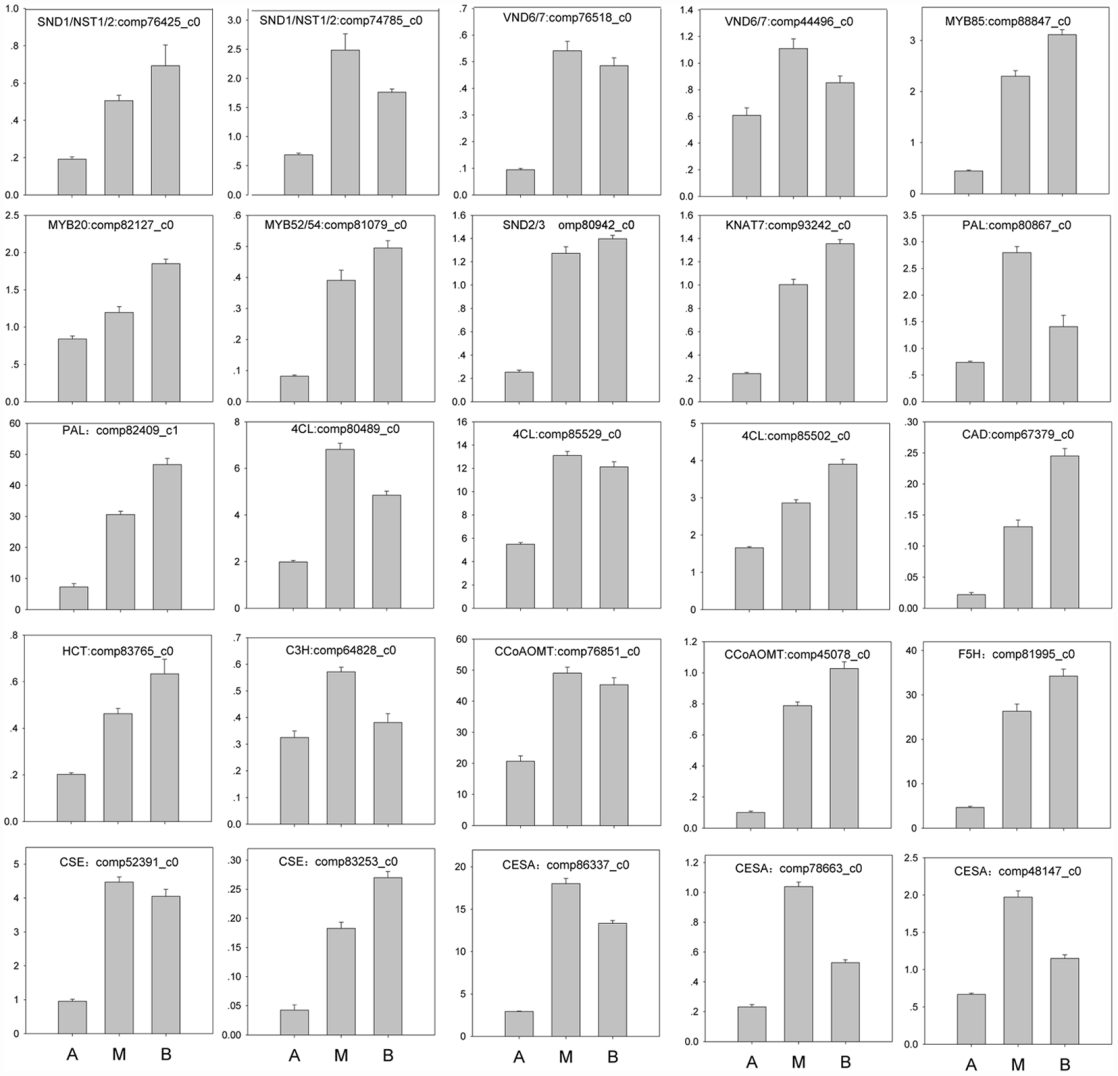
Expression of UniGenes related to transcription factors regulating SCW biosynthesis, lignin, or cellulose biosynthesis. Error bars on each column indicate SEs from three replicates. A, apical stem segment; M, middle stem segment; B, basal stem segment.

DEGs related to cell wall biosynthesis, including transcription factors and structural genes with expression levels higher in both basal and middle stem segments compared to apical stem segments, were examined for their expression patterns in the three stem segments representing different stages of xylogenesis. Genes for which no primers could be designed were excluded (Fig 8). Additionally, genes involved in cadambine, triterpenoid saponin or phytosterol biosynthetic pathways were also examined for their expression in roots, middle stem segments, young leaves, mature leaves, flowers, bark and cambium, with the exception of a few genes for which no primers could be selected (S4 Fig). As shown in Fig 8, all genes were expressed at a higher level in both middle and basal stem regions compared to the apical part of the stem, demonstrating a high correlation between RNA-seq and RT-qPCR data.

## Discussion

### *N*. *cadamba* transcriptome database by Illumina HiSeqTM 2000 sequence

It is easier to clone and characterize target genes in model plants such as Arabidopsis, rice, and poplar that have a fully sequenced genome and other available tools for genetic manipulation. However, to carry out similar work or any genetic improvement research, it is difficult to clone genes of interest in plants such as *N*. *cadamba* for which no genomic information is available. Despite the fact that the *N*.*cadamba* genome is not sequenced, RNA-Seq has been used to provide the sequences of expressed genes. In this report, 55,171 UniGenes with intact ORFs were predicted using the Getorf software (http://emboss.sourceforge.net/apps/cvs/emboss/apps/getorf.html, Table 2). Thus, genes of interest from *N*. *cadamba* can directly be cloned through the database and/or together with 5’ or 3’RACE.

Among the 55,432 unique sequences, 28,945 UniGenes (52.2%) had no functional annotation in the present study (Table 2, S9 File). Thus, half of the UniGenes did not match known protein families in the five public protein databases. Therefore, we consider them to represent unknown protein families, indicating that novel information was discovered in our Illumina data sets, in particular the 1,649 UniGenes without functional annotation among the DEGs. There were 49,230 UniGenes (88.8%) that comprise a group that did not show differential expression between the three stem segments (S9 File). The large number of genes with common expression levels in the three stem region transcriptomes suggests that the majority of the transcripts are involved in basal cellular metabolism processes and that the distinctive characteristics of each xylem region are derived from differences in the expression levels of a relatively small number of genes.

### Regulation of secondary metabolism during xylogenesis

The major goal of the present study was to carry out a preliminary screen for key genes involved in *N*. *cadamba* xylogenesis. Several studies in Arabidopsis have identified a network of transcription factors regulating the expression of numerous genes directly involved in the biosynthesis of secondary cell walls [13, 14]. The master switches for fibre (SND1, NST1, NST2) [47, 57, 69], protoxylem (VND7) and metaxylem (VND6) [58] differentiation are thought to initiate the transcriptional network for secondary cell wall formation by binding to SNBE (Secondary wall NAC-Binding Element) regulatory regions in the promoters of target genes. These include the two core transcription factors, MYB46 and MYB83, whose promoters each contain several SNBE promoter elements and are thought to be direct targets of secondary cell wall NAC genes [48, 49, 53, 64]. The MYB46/83 node activates the expression of numerous other transcription factors, whose activity amplifies the transcriptional network and thereby promotes lignin, cellulose and/or hemicelluloses biosynthesis [48, 49, 53, 54]. In this study, most of these transcription factor genes were up-regulated during xylogenesis in *N*. *cadamba* (Figs 4 and 8). These data indicate that these transcription factors are involved in secondary cell wall biosynthesis during xylogenesis in *N*. *cadamba*.

UniGenes in several metabolic pathways involved in secondary wall formation and/or maintenance showed differential expression between different regions of the stem (Figs 5 and 6). Transcription factors MYB58, MYB63, or MYB85 specifically regulate lignin biosynthesis/deposition, with the MYB58 and MYB63 directly activating expression of nearly all the genes involved in the lignin biosynthetic pathway. Both MYBs are thought to bind at conserved AC regulatory elements found upstream of the majority of lignin biosynthetic genes [50, 52, 54], with the exception that MYB103 is required for F5H expression and syringyl lignin biosynthesis [14]. Transcription factors SND2, SND3, and MYB103 are able to activate the promoters of genes involved in cellulose biosynthesis, such as CesA8, while MYB52 andMYB54 activate gene promoters involved in cellulose and xylan biosynthesis. In this study, the expression levels of these downstream transcription factors and some UniGenes in most metabolic steps in lignin and cellulose biosynthesis were similar to those of the upstream transcription factors. Expression levels of these transcription factors were significantly higher in both middle and basal stem segments of *N*. *cadamba*, compared to the apical stem segment (Figs 4–6, S12–S14 Files). Our previous study has found that microsomes isolated from the middle and basal stem segments exhibit the highest activity of UDP-xylsynthase and xylosyltransferase, and higher expression of genes related to heteroxylan biosynthesis compared to the apical segment of *N*. *cadamba* stem [20]. Overall, these data will be beneficial to further understanding the regulatory networks involved in secondary cell wall formation.

### Genes involved in the cellulose/monolignol biosynthetic pathway

The UniGenes comp86965_c0 (82% identity with AtCesA1 by BLASTx), and comp52742_c0, comp86567_c0 and comp86567_c1 (high identity with AtCesA2/6/9 by BLASTx) exhibited higher expression levels in apical stem segments compared to the middle and basal stem segments (Fig 5, S13 File). Vascular bundles in the apical stem segment are formed from procambial cells and consist of primary xylem tissues [20], consistent with expression of these *CesA*s, which are proposed to be involved in primary cell wall synthesis based on their homologies with *A*. *thaliana* CesAs [5]. However, the differentially expressed UniGenes, comp86337_c0 (82% identity with AtCesA7 by BLASTx) and comp78663_c0 (73% identity with AtCesA4 by BLASTx) exhibited higher expression in both middle and basal stem segments compared to apical stem segments (Fig 5, S13 File). Furthermore, the amount of secondary xylem increased in both middle and basal stem segments [20]. This was primarily due to the activity of CesAs involved in secondary cell wall biosynthesis as reported in the case of *A*. *thaliana*, where CesA complex enzymes CesA4, CesA7 and CesA8 are required for cellulose synthesis [4]. All these observations indicate that the differentially expressed UniGenes are required for primary or second cell wall cellulose biosynthesis during stem development in *N*. *cadamba*.

In the monolignol biosynthetic pathway, the expression of 1–3 members of gene families (PAL, 4CL, HCT, C3H, CSE, CCoAOMT, F5H and CAD) increased in both the M and B libraries as compared to the A library (Fig 6, S14 File). The expression of these genes corresponded with our earlier cell wall component analysis of apical, middle and basal stem segments in *N*. *cadamba* showing that lignin levels were higher in the middle and basal stem segments compared to the apical segment [20]. These observations indicate that these genes are the main transcripts and strongest candidates for involvement in lignin biosynthesis in *N*. *cadamaba*. Furthermore, the number of *N*. *cadamba* UniGenes and the number of genes found in the *P*. *trichocarpa* [65] and *E*. *grandis* [3] genomes that encode each one of the eleven key enzymes involved in the lignin biosynthesis pathway were more than the number found in the *A*. *thaliana* TAIR database (Table 4). These comparisons suggest that lignin biosynthesis in trees is more complex and requires more genes.

### The new genetic model tree for xylogenesis

Unlike the model plant Arabidopsis, tree species such as *N*. *cadamaba* accumulate higher amounts of secondary xylem. There are many more genes related to lignin biosynthesis in trees, compared to Arabidopsis (Table 4), which forms relatively little lignin rich in G units [70]. In contrast, trees typically have roughly equal amounts of G and S units comprising their lignin. This suggests that although Arabidopsis has been considered an excellent genetic model for the study of lignin biosynthesis in trees [71], there are a number of disadvantages. These include fewer xylem cell types, small plant size, and an annual growth habit, which means that studies into seasonal variation of xylem differentiation, dormancy, and cambial aging process cannot be carried out [72]. *N*. *cadamba* is a fast-growing tree for which a highly efficient *in vitro* regeneration system has been successfully established [24]. Furthermore, the CRISPR-Cas9 system for genome engineering has been established and applied widely to elucidate the functional organization of the genome at the systems level, and establish causal linkages between genetic variations and biological phenotypes [73]. These factors suggest that *N*. *Cadamba* might be established as a model plant for cell wall biosynthesis and wood development studies in the future by adopting new genetic technologies.

## Conclusions

In this study, we have conducted the first large-scale analysis of the *N*. *cadamba* transcriptome and identified several genes responsible for *N*. *cadamba* xylogenesis using Illumina paired-end sequencing technology. With DEG profiling, our results have provided a vast amount of information about genes that are differentially expressed during xylogenesis. 1,634 UniGenes exhibited significantly higher expression levels in the basal and middle stem segments compared to the apical stem segment. They included *NAC* and *MYB* transcription factors related to secondary cell wall biosynthesis, genes related to most metabolic steps of lignin biosynthesis and *CesA* genes involved in cellulose biosynthesis. Further analysis of the generated transcriptome dataset will provide new insights into molecular mechanisms of wood formation in fast-growing trees.

## Supporting Information

S1 Fig GO categories of the UniGenes and DEGs. (TIF)

S2 Fig COG categories of the UniGenes. (TIF)

S3 Fig COG categories of the DEGs. (TIF)

S4 Fig Expression of UniGenes related to cadambine, triterpenoid saponin and phytosterol biosynthesis. Error bars on each column indicate SEs from three replicates. R, root; S, middle stem segment; L, young leaf; ML, mature leaf; F, flower; B, bark; C, cambium.
(TIF)

S1 File Cellulose, mannan and monolignol biosynthesis-related protein family members from *A*. *thaliana*. (XLSX)

S2 File Primers for RT-qPCR. (XLSX)

S3 File Number of UniGenes matching the 17 top species using BLASTx in the nr database. (XLSX)

S4 File Number of UniGenes enriching in each GO category. (XLS)

S5 File UniGenes annotated with the category of cellulose biosynthetic process (GO:0030244), lignin biosynthetic process (GO:0009809), xylan biosynthetic process (GO:0045492), glucuronoxylan biosynthetic process (GO:0010417) and mannan synthase activity (GO:0051753), respectively. (XLSX)

S6 File Number of Unigenes classified into each group in COG annotation. (XLS)

S7 File Pathway categories of the UniGenes. (XLSX)

S8 File UniGenes annotated with the phenylpropanoid biosynthesis pathways (Ko00940) and starch and sucrose metabolism (ko00500). The color red, green, blue and violet indicate UniGenes participating in lignin, cellulose, mannan and heteroxylan biosynthesis process, respectively.
(XLSX)

S9 File UniGenes identified as DEGs and with expression profiles where the expression abundance in the M and B libraries was higher than in the A library in DEGs, and UniGenes without annotation. (XLSX)

S10 File DEGs for each expression profile. (XLSX)

S11 File DEGs and UniGenes with expression profiles where the expression abundance in the M and B libraries was higher than in the A library in DEGs, annotated with the cellulose biosynthetic process (GO:0030244) category, lignin biosynthetic process (GO:0009809), xylan biosynthetic process (GO:0045492), glucuronoxylan biosynthetic process (GO:0010417) and mannan synthase activity(GO:0051753), respectively. The color red and green indicate the UniGenes from DEGs and UniGenes with the expression profile showing that the expression abundance in the M and B libraries was higher than in the A library in DEGs, respectively.
(XLSX)

S12 File Summary for transcription factors, MYB and NAC UniGenes in *N*. *cadamba*. The color red and blue indicate the UniGenes identified as DEGs and UniGenes with expression profiles where the expression abundance in the M and B libraries was higher than in the A library in DEGs, respectively.
(XLSX)

S13 File Detailed protein names, annotation and RNA-Seq expression data of Unigenes participating in cellulose and mannan biosynthesis. (XLSX)

S14 File Detailed protein names, annotation and RNA-Seq expression data of Unigenes participating in lignin biosynthesis. The color green and red indicate the UniGenes from DEGs and UniGenes with an expression profile showing that abundance in the M and B libraries was higher than in the A library in DEGs, respectively.
(XLSX)

S15 File Detailed protein names, annotation and RNA-Seq expression data of Unigenes participating in cadambine, triterpenoid saponin and phytosterol biosynthesis. (XLSX)

S1 Table Summary for DEGs. * indicates M as control sample and A as test sample.
(DOCX)
