# Supplementary figures and images for: Transcriptomic Analysis of Multipurpose Timber Yielding Tree Neolamarckia cadamba during Xylogenesis Using RNA-Seq
Source: PLoS One. 2016 Jul 20;11(7):e0159407. doi: 10.1371/journal.pone.0159407 (PMC4954708; doi:10.1371/journal.pone.0159407)

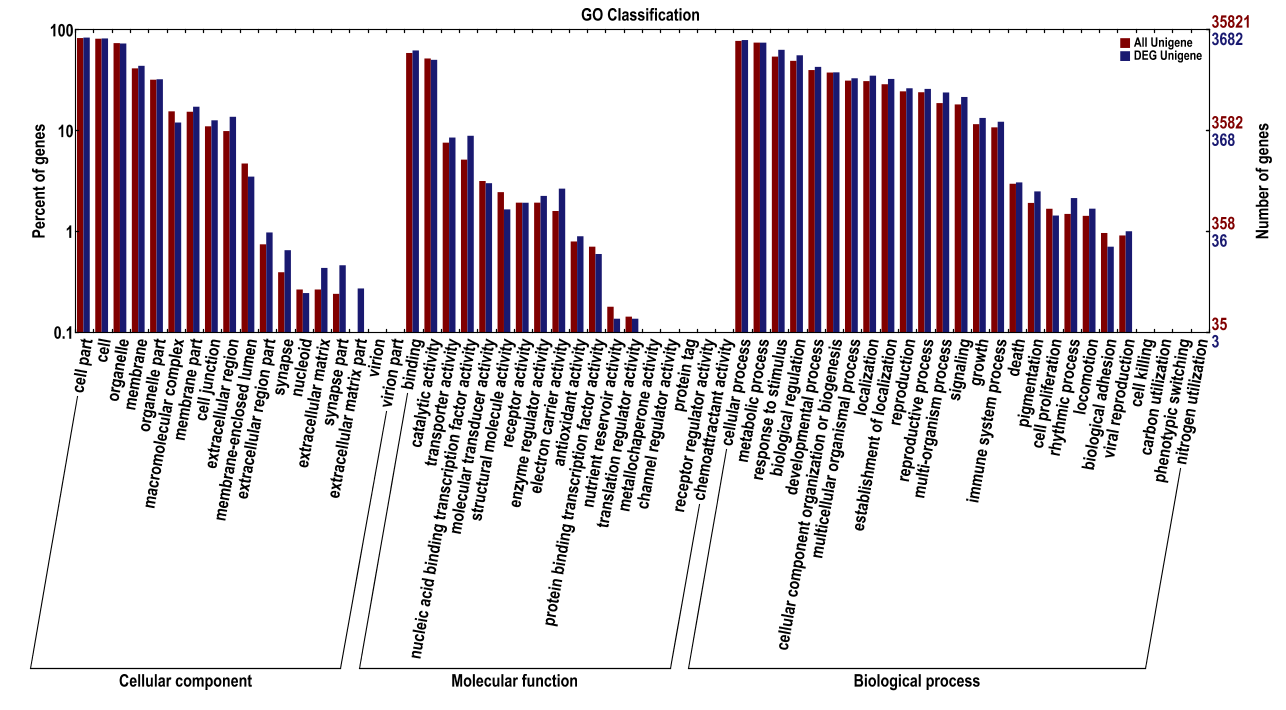

Supplement: S1 Fig — (TIF) [file pone.0159407.s001.tif]

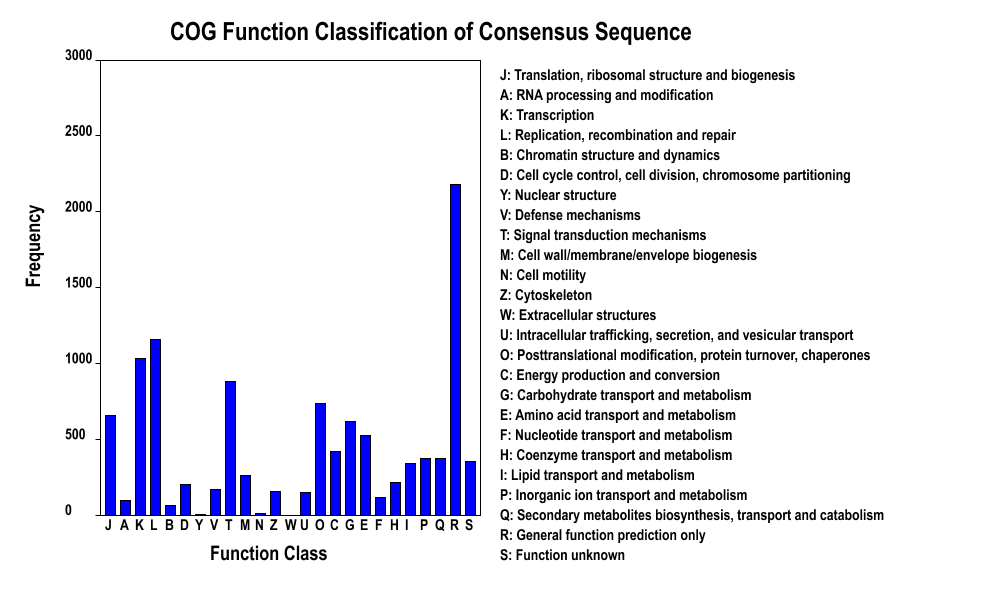

Supplement: S2 Fig — (TIF) [file pone.0159407.s002.tif]

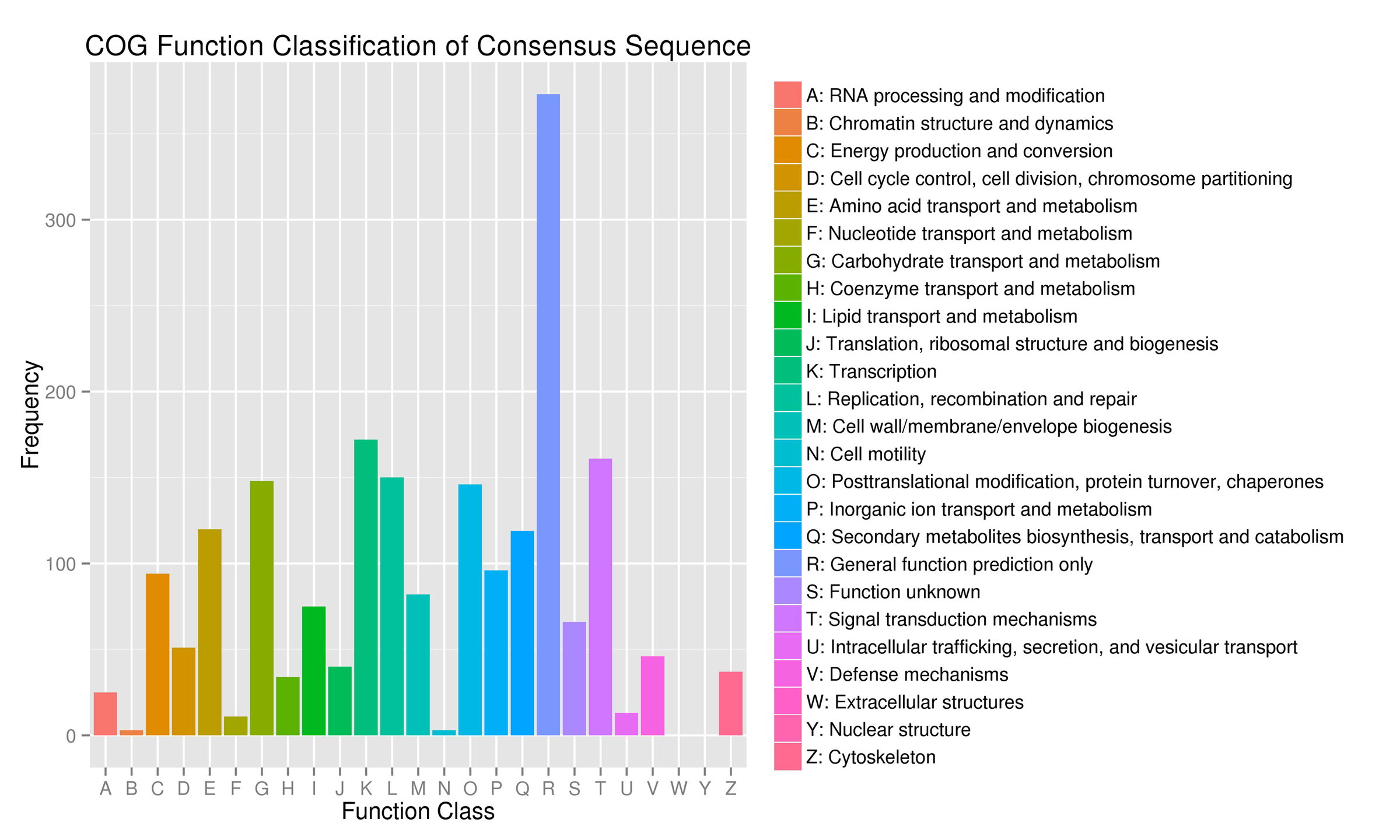

Supplement: S3 Fig — (TIF) [file pone.0159407.s003.tif]

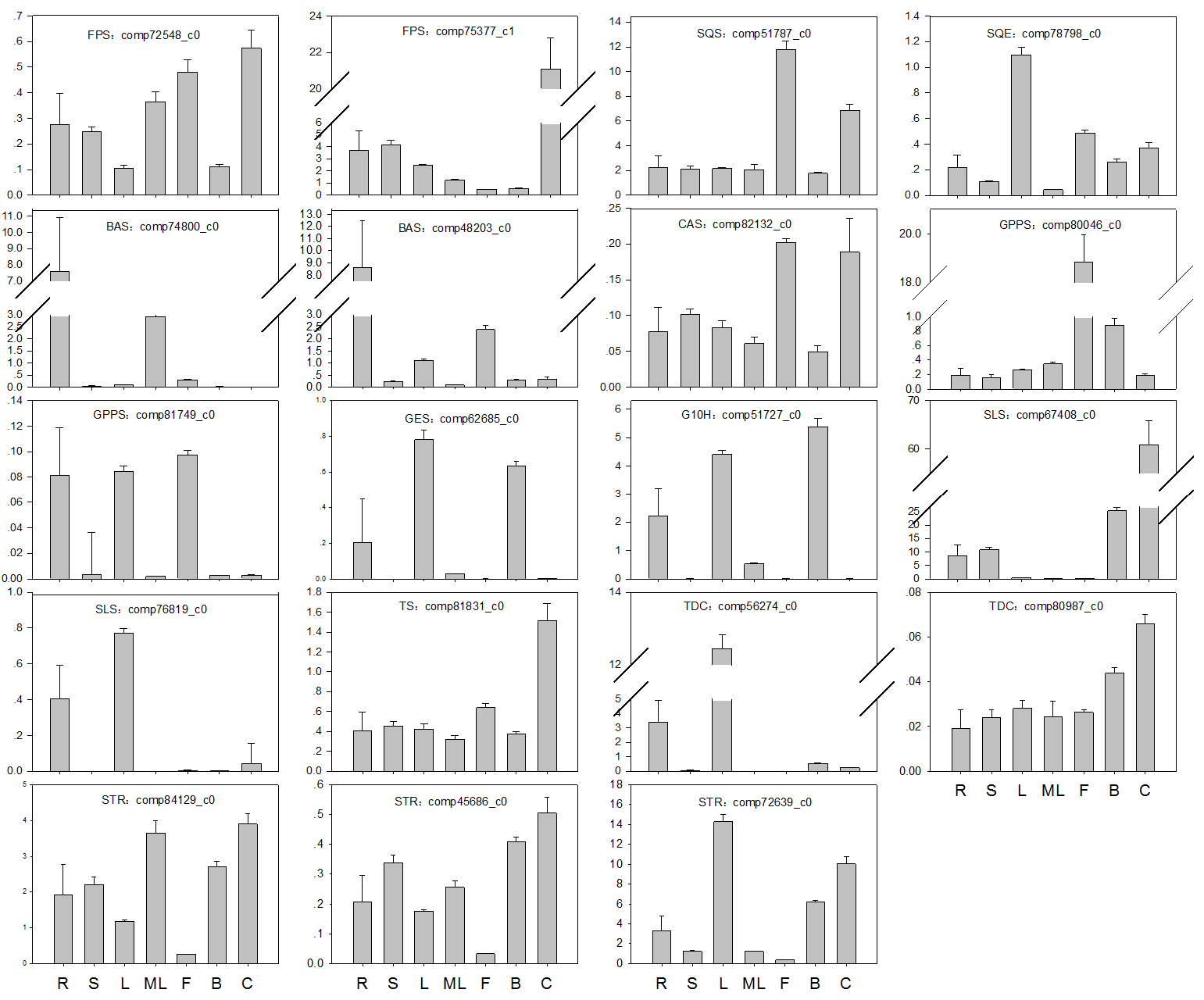

Supplement: S4 Fig — Error bars on each column indicate SEs from three replicates. R, root; S, middle stem segment; L, young leaf; ML, mature leaf; F, flower; B, bark; C, cambium. (TIF) [file pone.0159407.s004.tif]
