# Supplementary material for: Transcriptomic Analysis of Multipurpose Timber Yielding Tree Neolamarckia cadamba during Xylogenesis Using RNA-Seq
Source: PLoS One. 2016 Jul 20;11(7):e0159407. doi: 10.1371/journal.pone.0159407 (PMC4954708; doi:10.1371/journal.pone.0159407)
Supplement: S1 Table — * indicates M as control sample and A as test sample. (DOCX) [file pone.0159407.s020.docx]

| Type | Number | Up | Down |
| --- | --- | --- | --- |
| M_vs_A* | 3,293 | 2,218 | 1,075 |
| B_vs_A | 3,614 | 2,448 | 1,166 |
| M_vs_B | 2,963 | 1,357 | 1,606 |
